# Pellino1 regulates neuropathic pain as well as microglial activation through the regulation of MAPK/NF-κB signaling in the spinal cord

**DOI:** 10.1186/s12974-020-01754-z

**Published:** 2020-03-14

**Authors:** Lijuan Wang, Cui Yin, Tianya Liu, Mannan Abdul, Yan Zhou, Jun-Li Cao, Chen Lu

**Affiliations:** 1 School of Anesthesiology, Jiangsu Province Key Laboratory of Anesthesiology, Xuzhou Medical University, Xuzhou, 221002 China; 2 Department of Pharmacy, The Affiliated Hospital of Xuzhou Medical University, Xuzhou Medical University, Xuzhou, 221002 China; 3 Department of Neurology, The Affiliated Hospital of Xuzhou Medical University, Xuzhou Medical University, Xuzhou, 221002 China; 4 Xuzhou Medical University, 209 Tongshan Road, Xuzhou, 221004 Jiangsu China

**Keywords:** Neuropathic pain, Pellino1, Microglial activation, MAPK signaling, NF-κB, Neuroinflammation

## Abstract

**Background:**

Spinal cord microglia plays a crucial role in the pathogenesis of neuropathic pain. However, the mechanisms underlying spinal microglial activation during neuropathic pain remain incompletely determined. Here, we investigated the role of Pellino1 (Peli1) and its interplay with spinal microglial activation in neuropathic pain.

**Methods:**

In this study, we examined the effects of Peli1 on pain hypersensitivity and spinal microglial activation after chronic constriction injury (CCI) of the sciatic nerve in mice. The molecular mechanisms involved in Peli1-mediated hyperalgesia were determined by western blot, immunofluorescence, quantitative polymerase chain reaction (qPCR), and enzyme-linked immunosorbent assay (ELISA). We utilized immunoprecipitation to examine the ubiquitination of tumor necrosis factor receptor-associated factor 6 (TRAF6) following CCI. In addition, we explored the effect of Peli1 on BV2 microglial cells in response to lipopolysaccharide (LPS) challenge.

**Results:**

We found that CCI induced a significant increase in the levels of Peli1, which was present in the great majority of microglia in the spinal dorsal horn. Our results showed that spinal Peli1 contributed to the induction and maintenance of CCI-induced neuropathic pain. The biochemical data revealed that CCI-induced Peli1 in the spinal cord significantly increased mitogen-activated protein kinase (MAPK) phosphorylation, activated nuclear factor kappa B (NF-κB), and enhanced the production of proinflammatory cytokines, accompanied by spinal microglial activation. Peli1 additionally was able to promote K63-linked ubiquitination of TRAF6 in the ipsilateral spinal cord following CCI. Furthermore, we demonstrated that Peli1 in microglial cells significantly enhanced inflammatory reactions after LPS treatment.

**Conclusion:**

These results suggest that the upregulation of spinal Peli1 is essential for the pathogenesis of neuropathic pain via Peli1-dependent mobilization of spinal cord microglia, activation of MAPK/NF-κB signaling, and production of proinflammatory cytokines. Modulation of Peli1 may serve as a potential approach for the treatment of neuropathic pain.

## Introduction

Neuropathic pain is triggered by the damage to the peripheral nerve or central nervous system and characterized by increased sensitivity to innocuous and noxious stimulation [1–3]. At present, there is no effective therapy for neuropathic pain in the clinical setting which can provide progressive and complete treatment. Hence, for the evolution of a complete long-term treatment, there is a need for further investigations to explore the underlying mechanisms of neuropathic pain.

Numerous studies have demonstrated that microglial cells in the spinal cord play a vital role in the pathogenesis of neuropathic pain [3–7]. Nerve injury leads to a rapid microglial reaction along with intensive microgliosis in the spinal dorsal horn, including morphological alteration, proliferation, and upregulation of microglial markers [5, 8–10]. Various molecules and signaling pathways are involved in microglial activation at the level of the spinal cord during neuropathic pain, such as MAPK signaling and NF-κB [11–13]. Additionally, after nerve damage, the upregulation of proinflammatory cytokines including tumor necrosis factor α (TNF-α), interleukin 6 (IL-6), and interleukin 1β (IL-1β) released from activated microglial cells are involved in the sensitization of neurons via microglia-neuron interaction in the spinal cord [14, 15]. Notably, activation of p38 MAPK in spinal cord microglia, but not in neurons and astrocytes, contributes to neuropathic pain via the induction of proinflammatory cytokines [16, 17]. Although there are several mechanisms that were identified previously, but the underlying mechanisms of spinal microglial activation in neuropathic pain is still not fully understood.

Peli1 molecules form a conserved family of E3 ubiquitin ligases [18]. Among different Peli, Peli1 is of particular interest because it is abundantly expressed in microglial cells upon LPS stimulation relative to other family members [19]. Recent studies have shown that Peli1 plays an important role in modulating NF-κB activation and MAPK signaling [19, 20]. In addition, Peli1 is required for ubiquitination of substrate proteins such as TRAF6 to regulate NF-κB activation and MAPK signaling [21, 22]. Moreover, Peli1 is emerging as a microglia-specific regulator to participate in the pathophysiological process of experimental autoimmune encephalomyelitis (EAE) [19], West Nile virus (WNV) encephalitis [23], and subarachnoid hemorrhage (SHA) [24], suggesting causative involvement of Peli1 in neuroinflammation. However, it is unclear whether Peli1 would modulate spinal neuroinflammation in neuropathic pain.

In the present study, we investigated the role of Peli1 and its related regulatory mechanisms involved in neuropathic pain. Here, we found that spinal Peli1 was responsible for the induction phase and even the maintenance phase of neuropathic pain. The upregulation of Peli1 evoked spinal microglial activation along with the production of TNF-α, IL-6, and IL-1β through MAPK phosphorylation and NF-κB activation after CCI. We addressed that Peli1 in BV2 microglial cells significantly triggered inflammatory reactions in response to LPS challenge. These findings highlight a new function of Peli1 in neuropathic pain, and therefore it may serve as a novel therapeutic target for neuropathic pain.

## Materials and methods

### Animals and CCI model

Male Kunming mice (25–30 g) were obtained from the Division of Laboratory Animal Resources at Xuzhou Medical University. All animal care and experimental protocols were approved by Xuzhou Medical University Animal Care and Use Committee. All procedures were performed in accordance with the Guide for the Care and Use of Laboratory Animals published by the National Institutes of Health (NIH Publication, 8th edition).

Chronic constriction injury was induced as previously described [25]. Briefly, mice (25–30 g) were anesthetized with intraperitoneal injection of sodium pentobarbital (60 mg/Kg). Following the skin incision, the left common sciatic nerve was exposed and 3 ligatures (6–0 silk) were placed around the nerve proximal to the trifurcation with about 1 mm between ligatures. The ligatures were tied loosely until a short flick of the ipsilateral hind limb was observed. Sham-operated mice were subjected to the same surgical procedure except for ligation of nerve. All animals recovered in pre-warmed cages.

### Lentiviral shRNA vectors and siRNAs administration

Three recombinant lentiviral vectors carrying Peli1 shRNA (ShRNA1: 5′-GGATTTATG CTGCAGGGTTTG-3′; ShRNA2: 5′-GGTGGTTGAATATACTCATGA-3′; shRNA3: 5′-GGTTCACAGAAGACTCCA AAC-3′) and the lentiviral vectors carrying scrambled shRNA (shScr: 5′-TTC TCC GAA CGT GTC ACG T-3′) were designed and packaged using pGLV3-GFP vector by GenePharma Corporation (Shanghai, China).

Peli1 siRNA (siPeli1: 5′-AUUUAUGCUGCAGGGUUUGTT-3′) was designed. Negative siRNA (siNeg: 5′-UUCUCCGAACGUGUCACGUTT-3′) was used as control. siRNAs were synthesized by GenePharma Corporation (Shanghai, China). The siRNA was dissolved in RNase-free water at 1 μg/μl and mixed with the transfection reagent branched polyethyleneimine (PEI; Sigma-Aldrich) and 10% glucose for 15 min at room temperature before use.

### Experimental design

To examine the effects of Peli1 on the induction of neuropathic pain, mice were given 10 μl Peli1 shRNA (2 × 10^8^ TU) or 10 μl scrambled shRNA (1 × 10^9^ TU) 3 days prior to the beginning of CCI by intrathecal injection. The dosages of shRNA were selected based on previous reports [26, 27]. Thermal hyperalgesia and mechanical allodynia were measured before and 3, 5, 7, 11, 14 days after CCI.

To evaluate the effects of Peli1 on established neuropathic pain behavior, 5 μg Peli1 siRNA or 5 μg negative siRNA was intrathecally administrated daily for 3 consecutive days during postoperative days 5–7. The dosages of siRNA were selected based on previous reports [28]. Thermal hyperalgesia and mechanical allodynia were measured before and 3, 5, 7, 9 days after CCI.

### Assessment of pain behaviors

Thermal sensitivity was determined as described previously [29]. Paw withdrawal latency (PWL) was measured using a Hargreaves radiant heat apparatus (IITC Life Science). The basal PWL was adjusted to 14 to 16 s with a cutoff of 20 s to prevent tissue damage.

Mechanical allodynia was assessed as described previously [30]. Mice in the plastic box were placed on an elevated metal mesh floor. The hind paws were stimulated with a series of von Frey hairs (0.16–2 g; Stoelting) applied perpendicularly to the central plantar surface. A sharp withdrawal of the paw indicated a positive response. The 50% paw withdrawal threshold (PWT) was determined using Dixon’s up-down method.

### Immunofluorescence

In brief, mice were sacrificed after CCI and perfused with 4% buffered paraformaldehyde via the ascending aorta. The L4-L5 spinal cord segments were removed, post-fixed at 4 °C, cryoprotected in 30% sucrose, and embedded in the cryostat (Sakura, 4583). The tissues were sectioned into 16 μm thick using the Vibratome (Leica). The sections were blocked with PBS containing 4% normal donkey serum (Jackson ImmunoResearch, 136110) for 1 h at room temperature and stained with the primary antibody at 4 °C overnight. After washing, the sections were incubated with the secondary antibody for 2 h at room temperature. The sections were then mounted using Prolong gold antifade reagent with DAPI (Invitrogen, P36931). All images were taken using a confocal microscope (FluoView1000; Olympus). Quantitative analyses were calculated using the ImageJ software (NIH). The following antibodies were used: anti-Peli1 (1:100; Santa Cruz Biotechnology, sc271065), anti-GFAP (1:200; Santa Cruz Biotechnology, sc6170), anti-NeuN (1:1000; Cell signaling technology, 24307), anti-NeuN (1:1000; Millipore, ABN90), anti-Iba1 (1:500; Wako chemicals, 019-19741), anti-Iba1 (1:300; Abcam, ab48004), anti-p-p38 (1:1000; Cell signaling technology, 4511), anti-c-Fos (1:400; Cell signaling technology, 2250), anti-mouse IgG Alexa Fluor 488 (1:300; Abcam, ab150109), anti-goat IgG Alexa Fluor 555 (1:300; Abcam, ab150134), anti-goat IgG Alexa Fluor 488 (1:300; Abcam, ab150133), anti-rabbit IgG Alexa Fluor 594 (1:300; Invitrogen, A-11012), and anti-Guinea pig IgG Alexa Fluor 488 (1:200; Jackson ImmuneResearch, 706545148). For quantification of the number of immunopositive cells, we randomly selected four sections from each mouse. Cell counts were averaged to reflect the number of positive cells.

### Quantitative polymerase chain reaction (qPCR)

In brief, total RNAs were isolated with RNA isolator total RNA extraction reagent (Vazyme, R401-01) according to the manufacturer’s instructions. cDNA was synthesized using PrimeScipt RT Master Mix (Takara, RR036A). Ten microliters of qPCR reactions were prepared from 5 μl Premix Ex TaqII (Takara, RR820A), 0.5 μl primer (final concentration 10 nM), 2 μl DEPC water, and 2 μl cDNA. Reactions were run in a LightCycler 480 qPCR instrument (Roche) using the standard conditions 95 °C for 5 min, 40 cycles (95 °C for 15 s, 56 °C for 30 s, and 72 °C for 30 s) plus melting curve. Relative levels were quantified with the 2-ΔΔCT method that was normalized to the GAPDH. Sequences for all RT-qPCR primers are presented in Table 1.

**Table 1:** Primer sequences used for real-time polymerase chain reaction analysis

Gene name	Forward primer sequence (5'-3')	Reverse primer sequence (5'-3')
Peli1	CCTTGTCCATGTAAGTTTCTC	CAGAGTTCAGAAGTCTGGAAC
Iba1	GGATTTGCAGGGAGGAAAAG	TGGGATCATCGAGGAATTG
CD11b	TCCGGTAGCATCAACAACAT	GGTGAAGTGAATCCGGAACT
GFAP	GCACTCAATACGAGGCAGTG	GGCGATAGTCGTTAGCTTCG
iNOS	GGCAGCCTGTGAGACCTTTG	CATTGGAAGTGAAGCGTTTCG
TNF-α	CATCTTCTCAAAATTCGAGTGACAA	CCAGCTGCTCCTCCACTTG
IL-6	CACAGAGGATACCACTCCCAACA	TCCACGATTTCCCAGAGAACA
IL-1β	TGAGGCCCAAGGCCACAGGT	CATCTTCTCAAAATTCGAGTGACAA
CD86	ACGATGGACCCCAGATGCACCA	GCGTCTCCACGGAAACAGCA
Arg1	TTAGGCCAAGGTGCTTGCTGCC	TACCATGGCCCTGAGGAGGTTC
CD206	TCAGCTATTGGACGCGAGGCA	TCCGGGTTGCAAGTTGCCGT
Ym1	CACTGAACGGGGCAGGTCCAAA	ACCCCTGCCTGTGTACTCACCT
IL-10	GGCAGAGAACCATGGCCCAGAA	AATCGATGACAGCGCCTCAGCC
GAPDH	UUCUCCGAACGUGUCACGUTT	UUCUCCGAACGUGUCACGUTT

*Peli1* Pellino1, *Iba1* ionized calcium binding adapator molecule 1, *CD11b* cluster of differentiation 11b, *GFAP* glial fibrillary acidic protein, *iNOS* inducible nitric oxide synthase, *TNF-α* tumor necrosis factor α, *IL-6* interleukin 6, *IL-1β* interleukin 1β, *CD86* cluster of differentiation 68, *Arg1* arginase 1, *CD206* cluster of differentiation 206, *Ym1* chitinase-like protein 3, *IL-10* interleukin 10, *GAPDH* glyceradehyde-3 phosphate dehydrogenase

### Western blot

Western blot was performed as described previously [31]. Briefly, mice were given Peli1 shRNA or scrambled shRNA 3 days prior to the beginning of CCI by intrathecal injection. After 7 days of CCI, whole proteins were harvested from the ipsilateral spinal tissues at L4-L5 segments using lysis buffer with protease and phosphatase inhibitor cocktails. The protein concentrations were measured using BCA protein assay kit (Thermo Scientific, 23225). Whole lysates were separated with SDS-PAGE and transferred onto PVDF membranes (Millipore, IPV H00010). The PVDF membranes were incubated with the primary antibodies, including anti-Peli1 (1:100; Santa Cruz Biotechnology, sc-271065), anti-p-ERK (1:1000; Cell signaling technology, 4370), anti-p-p38 (1:1000; Cell signaling technology, 4511), anti-p-JNK (1:1000; Cell signaling technology, 4668), anti-ERK (1:1000; Cell signaling technology, 4695), anti-p38 (1:1000; Cell signaling technology, 8690), anti-JNK (1:1000; Cell signaling technology, 9252), anti-p-NF-κB p65 (1:1000; Cell signaling technology, 3033), anti-NF-κB p65 (1:500; Affinity, AF5006), and anti-GAPDH (1:1000; Proteintech, 60,004–1-1 g), respectively, followed by incubation with peroxidase-conjugated secondary antibodies (Cell signaling technology). The membrane was developed by ECL substrate (Thermo Scientific, 32106) and exposed by the ChemiDoc XRS system with Image Lab software (Bio-rad). The intensity of blots was quantified using the ImageJ software (NIH).

### Enzyme-linked immunosorbent assay (ELISA)

According to the instructions of the manufacturer, the levels of proinflammatory cytokines (TNF-α, IL-6, and IL-1β) in the ipsilateral spinal tissues or conditioned media from BV2 cells were examined using ELISA kit (Invitrogen, 88-7324-22, 88-7064-88, and 88-7013-88).

### Immunoprecipitation

Cellular proteins were extracted and incubated with 2 μg of anti-TRAF6 antibody (1:100; Santa Cruz Biotechnology, sc-8409) overnight at 4 °C, followed by incubation with 30 μl protein A/G agarose beads (Santa Cruz Biotechnology, sc-2003) overnight at 4 °C, as described previously. The precipitations were then washed three times with lysis washing buffer and added with loading buffer. After SDS-PAGE, the membrane was subjected to western blot with anti-ubiquitin antibody (1:1000; Cell signaling technology, 5621).

### In vitro experiments

BV2 microglial cells were cultured in Dulbecco modified eagle medium (DMEM) (HyClone, SH30022.01) supplemental with 10% fetal bovine serum (FBS) (Gibco, A3160902) and 100 U/ml penicillin (Invitrogen, 10378016 s) at 37 °C in a humidified atmosphere with 5% CO_2_. The following experiments were performed. First, BV2 cells were treated with or without lipopolysaccharide (LPS, Sigma-Aldrich, L2880) at concentrations of 10 ng/ml, 100 ng/ml, and 1000 ng/ml for 2 h. Cell lysates were used to examine the levels of Peli1 after LPS treatment. Second, to examine the direct effect of Peli1 on microglia, 75 μm Peli1 siRNA or 75 μm negative siRNA was transfected into BV2 cells using RNAifectin (Applied Biological Materials, G073) under serum-free medium. After 8 h of incubation, the growth medium was replaced and the cultures were continued for 16 h before the assays were performed. BV2 cells were then stimulated with 100 ng/ml LPS for 2 h. Additionally, BV2 microglial cells were transduced with Peli1 shRNA or scrambled shRNA for 72 h before stimulated with LPS. Cell lysates were prepared for evaluation of MAPK signaling and NF-κB activation. Culture media were collected and centrifuged for measurement of TNF-α, IL-6, and IL-1β concentration using ELISA kits. Total RNAs were isolated for detection of correlation between Peli1 and microglial polarization using qPCR. Sequences for all qPCR primers are presented in Table 1. The activation of BV2 microglial cells was determined using immunofluorescence staining. Fifth, BV2 cells migration capacity was examined using the scratch assay. BV2 cells were seeded into 6-well plate and transfected with Peli1 siRNA or negative siRNA for 24 h as a confluent monolayer. Cells were then scratched with 200 μl tips and stimulated with 100 ng/ml LPS. Migration of cells in the wound area was photographed at 0 h and 24 h after injury. The percent closure of the wound was analyzed using ImageJ (NIH).

### Statistical analysis

All parameters were presented as Mean ± SEM. Comparisons of data between groups were followed using either Student’s *t* test or one-way analysis of variance (ANOVA), followed by Tukey’s post hoc test for multiple range tests. Time-series data were analyzed with repeated measurement two-way ANOVA. The difference was considered significant as *p* value is less than 0.05.

## Results

### Peli1 is increased in spinal cord after CCI

**Fig. 1:**
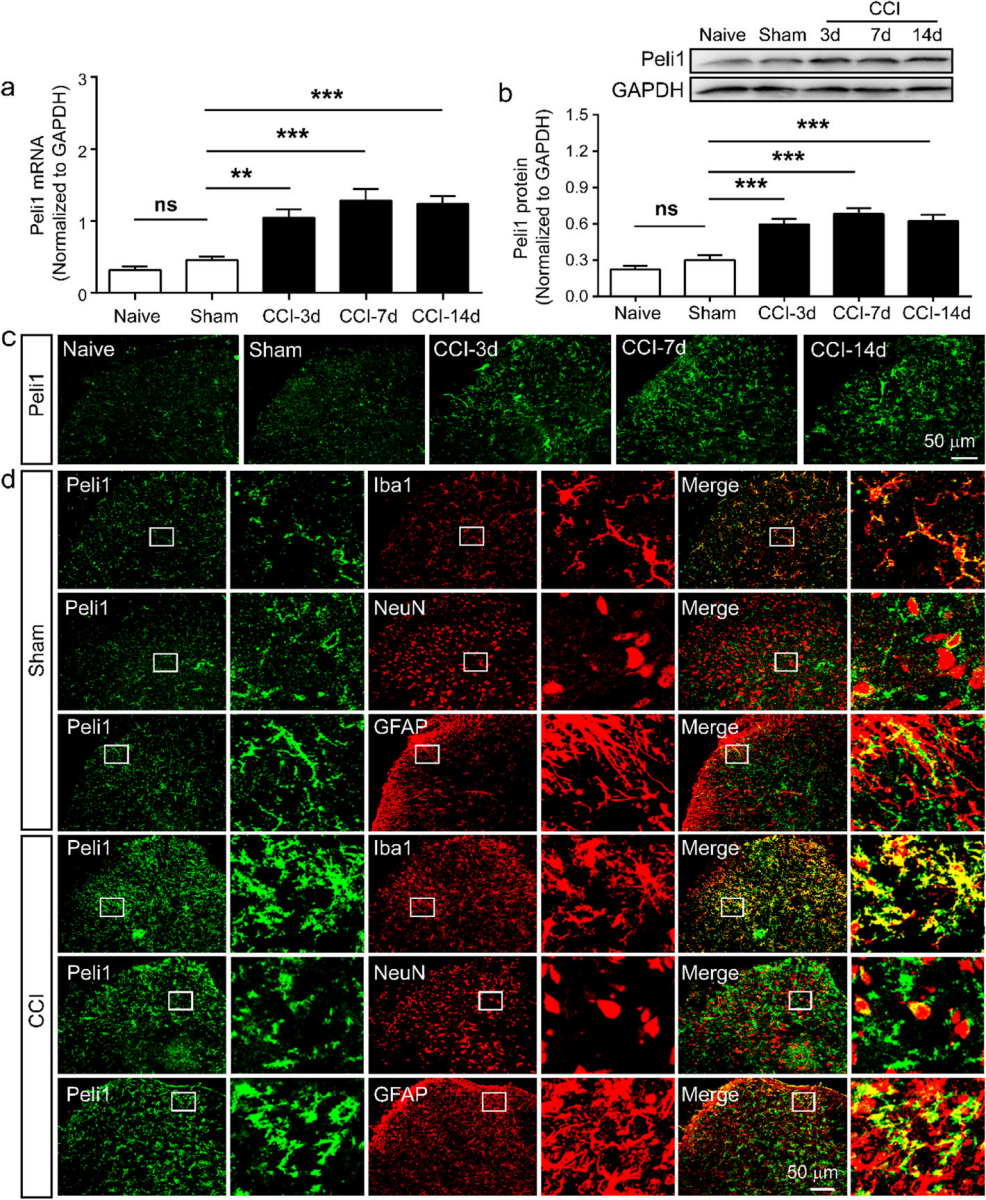
Peli1 is increased after CCI and mainly expressed in spinal cord microglia. **a** The time course of Peli1 mRNA expression in the ipsilateral spinal cord in naive, sham, and CCI mice. CCI increased Peli1 mRNA levels at 3 days, 7 days, and 14 days (*n* = 6). One-way ANOVA (*F*_4,25_ = 18.0). **b** Quantitative western blot analysis showing the increase of Peli1 protein expression in the ipsilateral spinal cord on days 3, 7, and 14 after CCI (*n* = 6). One-way ANOVA (*F*_4,25_ = 22.8). **c** Representative images of Peli1 immunofluorescence in the L5 ipsilateral dorsal horn (scale 50 μm, *n* = 4). **d** Representative images of subcellular colocalization of Peli1 in the ipsilateral spinal dorsal horn with a high magnification image (boxed). Double immunofluorescence staining showing Peli1 (green) is primarily co-staining with microglial cells (Iba1, red), rarely with neurons (NeuN, red), and astrocytes (GFAP, red) (scale bar 50 μm, *n* = 4). Results are expressed as the Mean ± SEM. **p < 0.01, ***p < 0.001 compared with indicated group. ns statistically not significant

To explore the potential role of Peli1 in neuropathic pain, we examined Peli1 expression in the ipsilateral spinal cord at segments L4-L5 on day 3, 7, and 14 following CCI. Figure 1 a shows that Peli1 mRNA levels were persistently elevated by 1.3 fold at 3 days, 1.8 fold at 7 days, and 1.7 fold at 14 days after CCI, when compared with sham control. Western blot revealed that the protein levels of Peli1 were significantly increased on day 3 and sustained on day 14 after CCI (Fig. 1b). There was no significant difference in Peli1 expression between naive and sham-operated mice (Fig. 1a, b). As shown in Fig. 1 c, immunofluorescence further showed CCI-induced higher expression of Peli1 in the ipsilateral dorsal horn at 3, 7, and 14 days, but not in naive and sham control.

To define the type of cells expressing Peli1 in the spinal dorsal horn, we performed co-staining of Peli1 with neuronal or glial markers. As shown in Fig. 1 d, in sham-operated mice, Peli1 immunoreactivity (IR) was co-stained excessively with microglial marker Iba1, to a lesser extent, with astrocytic marker GFAP and neuronal marker NeuN. We also determined Peli1 cellular localization after CCI. Approximately 88.6% of Iba1-labeled microglia was positive for Peli1 in laminae I-III of the ipsilateral spinal dorsal horn following CCI. Peli1 staining was observed in a limited number of spinal astrocytes and neurons. About 24.5% of astrocytes and 20.4% of neurons expressed Peli1 in laminae I-III of the ipsilateral dorsal horn.

### Peli1 is required for pain hypersensitivity

**Fig. 2:**
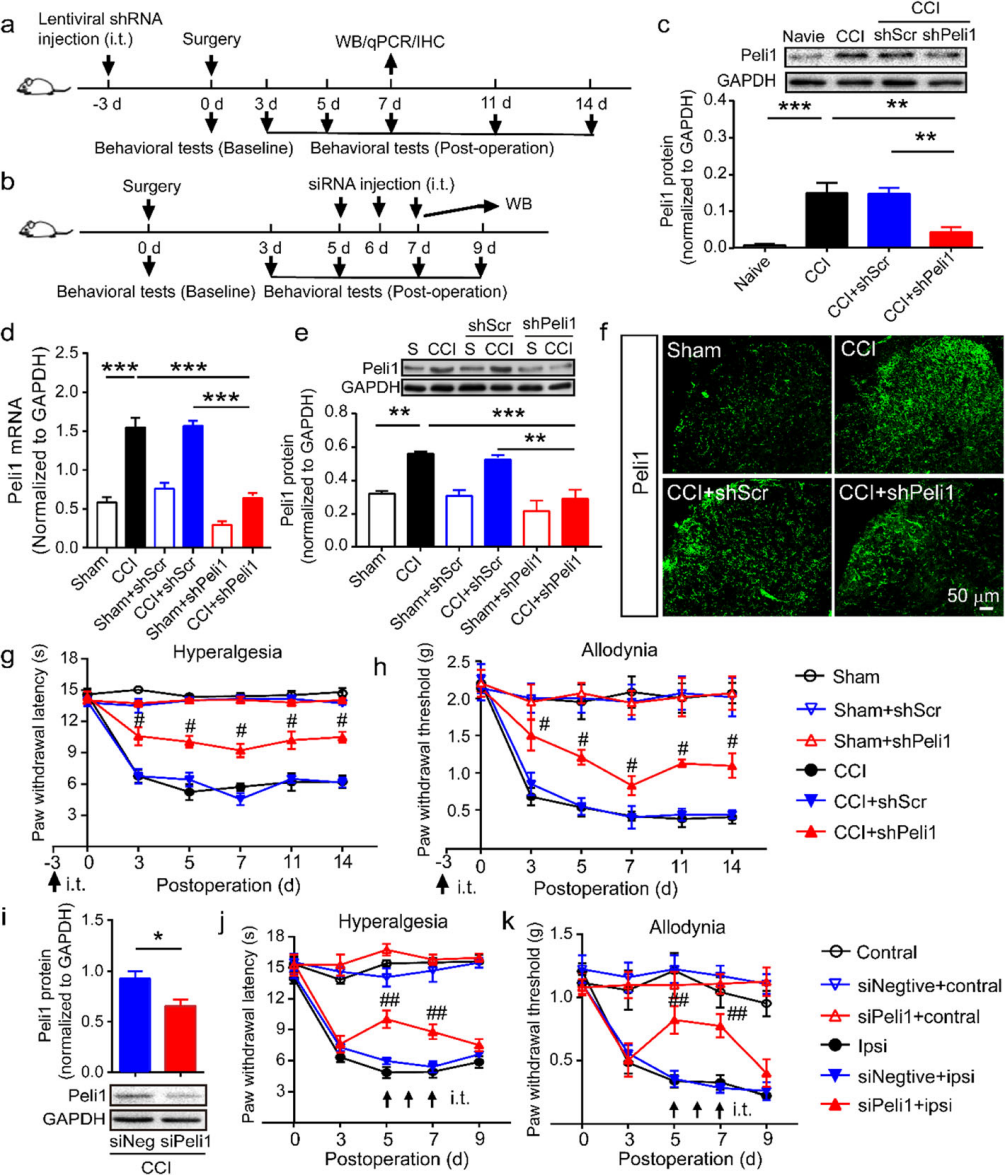
Spinal Peli1 contributes to the initiation and maintenance of neuropathic pain. **a**, **b** Timeline schematic of experimental paradigm. **c** Effective knockdown of Peli1 in vivo by lentiviral vectors carrying Peli1 shRNA (shPeli1) was examined using western blot. Lentiviral vectors carrying scrambled shRNA (shScr) were used as negative control (*n* = 4–6). One-way ANOVA (*F*_3,18_ = 13.3). **d**, **e** qPCR and western blot analysis showing the inhibitory effect of Peli1 shRNA on CCI-induced increases in Peli1 mRNA (**d**, *n* = 4–5) and protein (**e**, *n* = 4–5). One-way ANOVA [(**d**) *F*_5,20_ = 47.3; (**e**) *F*_5,20_ = 48]. **f** Immunofluorescence showing intrathecal injection of Peli1 shRNA eliminates CCI-induced increase in Peli1 immunoreactivity (scale bar 50 μm, *n* = 4). **g**, **h** Thermal sensitivity and mechanical allodynia were tested prior to surgery and on days 3, 5, 7, 11, and 14 after surgery. Peli1 shRNA (i.t., 3 days prior to the beginning of CCI, indicated by arrows) suppresses CCI-induced decreases in PWL (**g**) and PWT (**h**) values (*n* = 5). Repeated measures two-way ANOVA [(**g**) interaction *F*_25,144_ = 14.6; time *F*_5,144_ = 15.9; treatment *F*_5,144_ = 231.9 (**h**) interaction *F*_25,144_ = 3.5; time *F*_5,144_ = 21.7; treatment *F*_4,144_ = 76.5]. **i–k** Mice were given with Peli1 siRNA (siPeli1) and negative siRNA (siNeg) once daily from postoperative days 5 to 7 (indicated by arrows). Ipsilateral lumbar dorsal spinal tissues were harvested 4 h after the last siRNA injection for detection of Peli1 expression (**i**, *n* = 4). Intrathecal injection of peli1 siRNA reverses established thermal hyperalgesia (**j**) and mechanical allodynia (**k**) on day 5 and 7 following CCI (*n* = 5). Repeated measures two-way ANOVA [(**j**) interaction *F*_20, 120_ = 9.6; time *F*_4,120_ = 44.4; treatment *F*_5,120_ = 175.5 (**k**) interaction *F*_20,120_ = 4.2; time *F*_4,120_ = 19.8; treatment *F*_5,120_ = 51]. Results are expressed as the Mean ± SEM. **p < 0.01, ***p < 0.001 compared with indicated group. ^#^*p* < 0.05 compared with CCI and CCI plus scrambled shRNA. ^##^*p* < 0.05 compared with CCI and CCI plus negative siRNA

To investigate whether Peli1 in the spinal cord would participate in pain hypersensitivity, we determined the effect of Peli1 on the induction of neuropathic pain behaviors by knocking down Peli1 using lentiviral vectors carrying Peli1 shRNA (Fig. 2a). Lentiviral scrambled shRNA was used as a negative control. Mice were received intrathecal injection of Peli1 shRNA or scrambled shRNA 3 days before the beginning of CCI. We examined the GFP fluorescence to assess transfection efficiency in the lumbar spinal dorsal horn 6 days after the intrathecal injection. Immunofluorescent staining showed that GFP was expressed in neurons, astrocytes, and microglia (Fig. S1). On day 7 after CCI, the expression of Peli1 was examined to assess the knockdown efficiency using qPCR and western blot. Figure 2 c shows that CCI-induced Peli1 expression was much lower in Peli1 shRNA-injected mice (0.04 ± 0.01) than in CCI mice (0.15 ± 0.03). Moreover, intrathecal injection of Peli1 shRNA induced a significant decrease in Peli1 mRNA and protein levels by 59.3% and 54.1% in the ipsilateral spinal cord, respectively, when compared with intrathecal injection of scrambled shRNA (Fig. 2d, e). The results of immunofluorescence staining further supported that intrathecal injection of Peli1 shRNA led to a great decrease of Peli1 expression after CCI (Fig. 2f). Moreover, double immunofluorescence staining revealed co-staining of Peli1 with Iba1, NeuN, and GFAP indicting that a decreased expression of Peli1 in microglia, neurons, and astrocytes in Peli1 shRNA-treated mice after CCI (Fig. S2). After we validate the efficiency of shRNA in knocking down Peli1, we proceeded to perform the behavioral tests to examine whether knocking down Peli1 in the spinal cord would attenuate CCI-induced pain hypersensitivity. As shown in Fig. 2 g and h, CCI induced a significant reduction of PWL and PWT values. However, intrathecal injection of Peli1 shRNA dramatically alleviated thermal hyperalgesia and mechanical allodynia, which started at 3 days and was maintained for 14 days. Intrathecal injection of scrambled shRNA did not attenuate the severity of pain-related behaviors. As expected, there were no differences in the responses to thermal and mechanical stimulation were observed in sham-operated mice by either intrathecal injection of peli1 shRNA or scrambled shRNA. Moreover, no significant differences in the baseline of PWL and PWT values were observed among groups.

Knowing the role of spinal Peli1 in the initiation of CCI-induced pain hypersensitivity, we further examined whether blockade of spinal Peli1 would be sufficient to reverse pain sensitivity after an injury had already occurred. Peli1 siRNA or negative siRNA was intrathecally administrated daily for 3 consecutive days starting 5 days after CCI (Fig. 2b). We examined the efficiency of siRNA in knocking down Peli1 in the spinal cord. Figure 2 i shows that Peli1 siRNA resulted in significant downregulation of Peli1 expression. Figure 2 j and k show that repetitive intrathecal injection of Peli1 siRNA attenuated existing thermal hyperalgesia and mechanical allodynia on the ipsilateral side at 5 and 7 days after surgery. However, intrathecal injection of negative siRNA failed to have an analgesic effect on these pain-related behaviors. Neither Peli1 siRNA nor negative siRNA affected thermal or mechanical sensitivity on the contralateral side.

### Peli1 is necessary for CCI-induced microglial activation and the production of proinflammatory cytokines

**Fig. 3:**
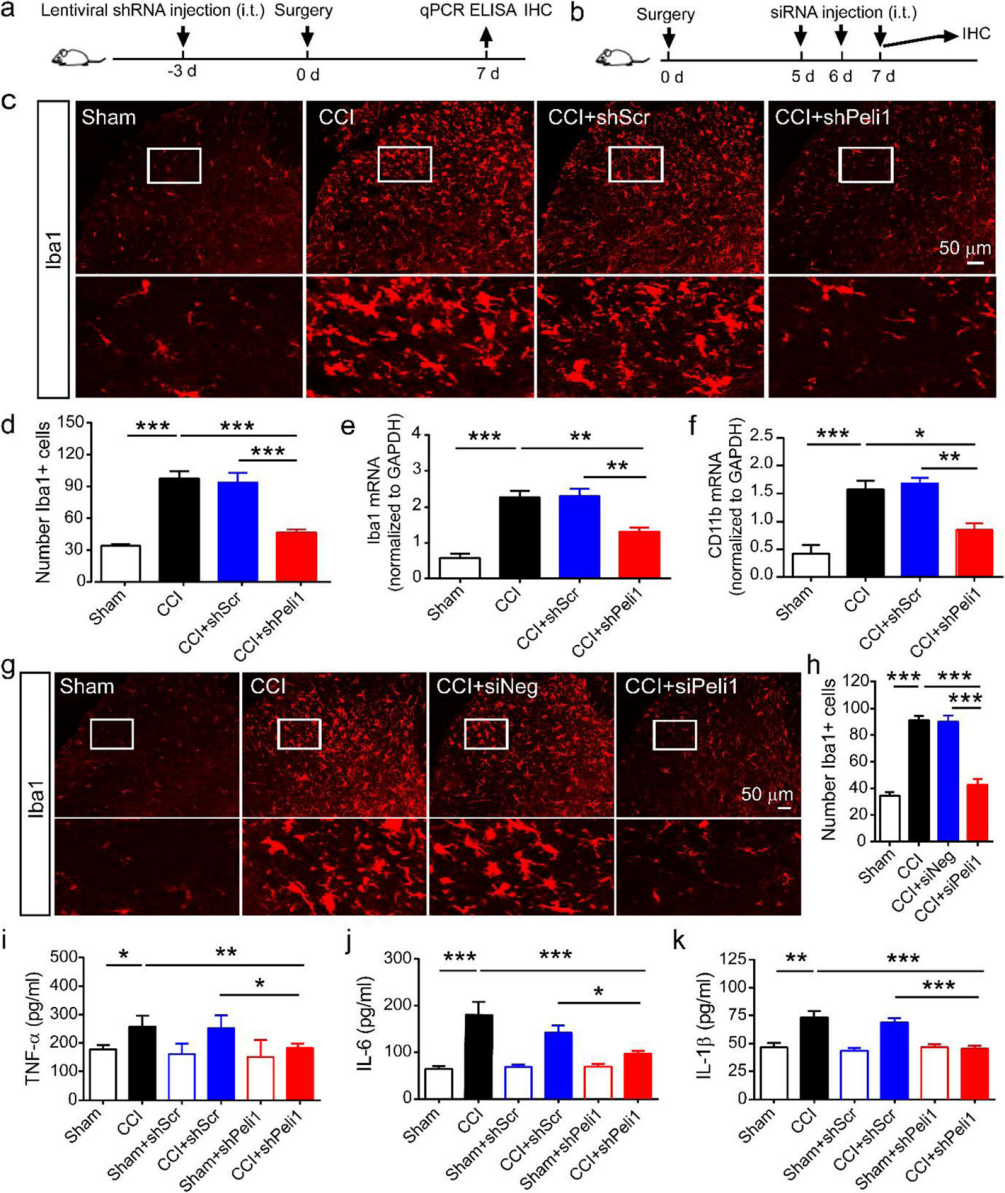
Blocking increased spinal Peli1 reverses CCI-induced microglial activation and the production of pro-inflammatory cytokines. **a**, **b** Timeline schematic of experimental paradigm. **c** Representative images of Iba1 immunofluorescence (red) on the ipsilateral side of dorsal horn with a high magnification image (boxed, scale bar 50 μm). **d** Qualitative data showing the inhibitory effect of Peli1 shRNA on the number of Iba1^+^ cells in the dorsal horn (*n* = 4). One-way ANOVA (*F*_3,12_ = 28.6). **e**, **f** Administration of Peli1 shRNA inhibits CCI-induced upregulation of the microglial marker Iba1 (**e**, *n* = 5–6) and CD11b (**f**, *n* = 5–6) mRNA expression in L4-L5 ipsilateral dorsal spinal cord. One-way ANOVA [(**e**) *F*_3,17_ = 28.3; (**f**) *F*_3,15_ = 18.2)]. **g**, **h** Representative images and quantitation of Peli1 immunofluorescence showing the inhibitory effect of Peli1 siRNA on established microglial activation on the ipsilateral side of the dorsal horn (scale bar = 50 μm, *n* = 4). One-way ANOVA (*F*_3,12_ = 62.7). **i–k** The levels of TNF-α (**i**, *n* = 4–5), IL-6 (**j**, *n* = 4–5), and IL-1β (**k**, *n* = 4–5) were measured on day 7 by ELISA. Spinal Peli1 knockdown inhibits CCI-induced TNF-α, IL-6, and IL-1β production in the ipsilateral dorsal horn. One-way ANOVA [(**i**) *F*_5,19_ = 10.9; (**j**) *F*_5,18_ = 11.9; (**k**) *F*_5,17_ = 13.9]. Results are expressed as the Mean ± SEM. *p < 0.05, **p < 0.01, ***p < 0.001 compared with indicated group

As microglial activation plays an important role in neuropathic pain, we sought to identify whether the decreasing spinal Peli1 would alter the status of microglia in the spinal dorsal horn after CCI. Microglial activation was demonstrated by the changes of number and morphology in Iba1-positive cells in lamina I-III layers of the dorsal horn. Mice were received intrathecal injection of Peli1 shRNA or scrambled shRNA 3 days before the beginning of CCI (Fig. 3a). As shown in Fig. 3 c and d, CCI triggered radical changes in the morphology and accumulation of Iba1 stained microglia in the spinal dorsal horn. By contrast, CCI induced these alterations of microglia were significantly attenuated by intrathecal injection of Peli1 shRNA, but not by scrambled shRNA (Peli1 shRNA, 46.5 ± 3.1; scrambled shRNA, 93.5 ± 9.0). We further evaluated microglial markers Iba1 and CD11b mRNA expression in the ipsilateral spinal cord. qPCR results showed that CCI robustly upregulated the levels of Iba1 and CD11b. Intrathecal injection of scrambled shRNA had no effect on this upregulation following CCI. By contrast, intrathecal injection of Peli1 shRNA significantly suppressed CCI-induced elevation of Iba1 and CD11b transcripts in the ipsilateral spinal cord (Fig. 3e, f). In addition, mice were received intrathecal injection of Peli1 siRNA or negative siRNA for 3 consecutive days starting 5 days after CCI (Fig. 3b). We observed that downregulation of Peli1 in the spinal cord resulted in a significant decrease in established microglial activation, as demonstrated by a reduction in the number of Iba1-positive cells, as well as morphological changes (Fig. 3g, h).

Previous studies have reported that microglial activation induces extensive production of proinflammatory cytokines, such as TNF-α, IL-6, and IL-1β, which are involved in the pathogenesis of neuropathic pain [32]. Hence, we evaluated the inhibitory effect of peli1 on TNF-α, IL-6, and IL-1β levels in the presence and absence of CCI by ELISA. As shown in Fig. 3i–k, CCI significantly enhanced the production of TNF-α, IL-6, and IL-1β compared with their respective sham controls. However, we observed that intrathecal administration of Peli1 shRNA significantly attenuated the production of TNF-α, IL-6, and IL-1β by 29.2%, 46.3%, and 38.7% in the ipsilateral spinal cord, respectively, in comparison with CCI control. Administration of scrambled shRNA did not reverse CCI-induced upregulation of TNF-α, IL-6, and IL-1β.

### Downregulation of Peli1 suppresses astrocyte activation after CCI

**Fig. 4:**
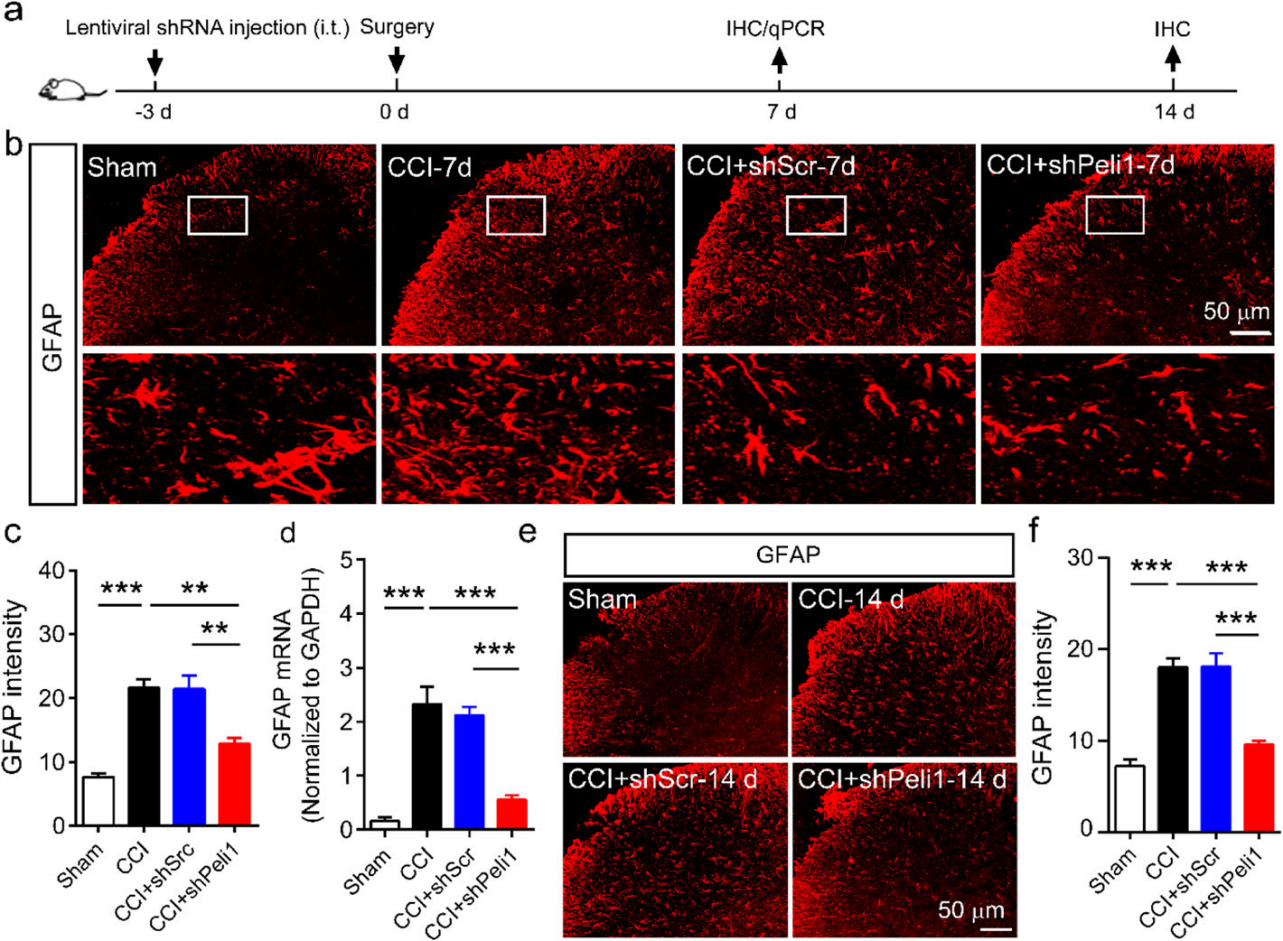
Spinal Peli1 knockdown inhibits CCI-induced astrocyte activation on the ipsilateral side of the dorsal horn. **a** Timeline schematic of experimental paradigm. **b** Representative images showing inhibitory effects of Peli1 shRNA on activation of astrocytes (GFAP) at 7 days after CCI with a high magnification image (boxed, scale bar, 50 μm). **c** Data summary of GFAP intensity (*n* = 4). One-way ANOVA (*F*_3,12_ = 26.3). **d** qPCR showing decreased GFAP mRNA levels in the ipsilateral spinal cord of Peli1 shRNA-treated mice on day 7 after CCI (*n* = 4–5). One-way ANOVA (*F*_3,15_ = 31.3). **e**, **f** Representative images and quantitation of GFAP intensity showing inhibitory effect of Peli1 shRNA on astrocytic activation at 14 days after CCI (scale bar 50 μm, *n* = 4). One-way ANOVA (*F*_3,12_ = 36.2). Results are expressed as the Mean ± SEM. **p < 0.01, ***p < 0.001 compared with indicated group

As activation of spinal astrocytes evokes pain hypersensitivity, we examined the effect of knocking down Peli1 in the spinal cord on astrocyte activation. Mice were intrathecally injected with Peli1 shRNA or scrambled shRNA 3 days before the beginning of CCI (Fig. 4a). As shown in Fig. 4 b and c, administration of scrambled shRNA resulted in a large increase of GFAP intensity on day 7 post-CCI. However, administration of Peli1 shRNA significantly attenuated CCI-induced astrocyte activation. We also found that CCI-induced elevation of GFAP mRNA in the ipsilateral spinal cord was significantly attenuated by Peli1 shRNA. (Fig. 4d). In addition, administration of Peli1 shRNA in days 14 post-induction of CCI significantly decreased GFAP intensity in comparison with administration of scrambled shRNA (Fig. 4e, f).

### Downregulation of Peli1 inhibits neuronal activation after CCI

**Fig. 5:**
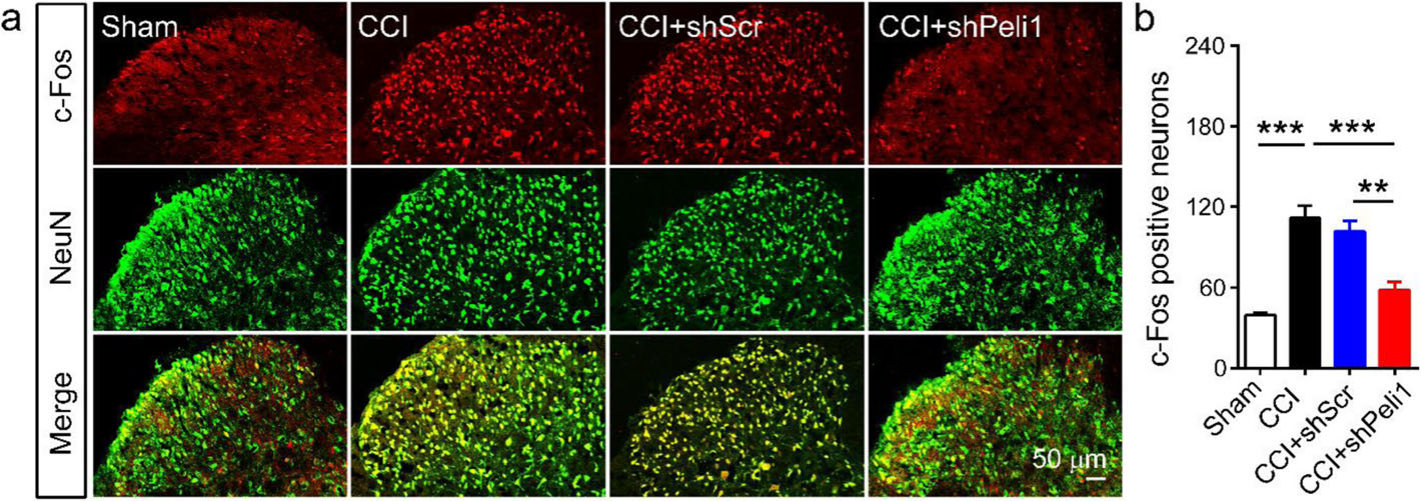
Spinal Peli1 knockdown inhibits CCI-induced neuronal activation on the ipsilateral side of the dorsal horn. **a** Representative images of c-Fos (red) and NeuN (green) in the ipsilateral dorsal horn. **b** Peli1 shRNA decreases the number of c-Fos positive neurons on day 7 after CCI (scale bar 50 μm, *n* = 4). One-way ANOVA (*F*_3,12_ = 25.2). Results are expressed as the Mean ± SEM. ***p < 0.001 compared with indicated group

We further determined the effect of Peli1 on neuronal activation in the ipsilateral dorsal horn. c-Fos, serving as a marker of neuronal activity, was detected on day 7 after CCI. As shown in Fig. 5 a and b, CCI caused a significant increase in the number of c-Fos positive neurons compared with sham control (111.8 ± 9.0 vs 39.5 ± 1.7). We found that intrathecal injection of Peli1 shRNA exhibited fewer c-Fos positive neurons (58.3 ± 6.0) in the ipsilateral dorsal horn, as compared to scrambled shRNA injection (101.5 ± 8.3).

### Downregulation of Peli1 attenuates CCI-induced MAPK phosphorylation and NF-κB activation

**Fig. 6:**
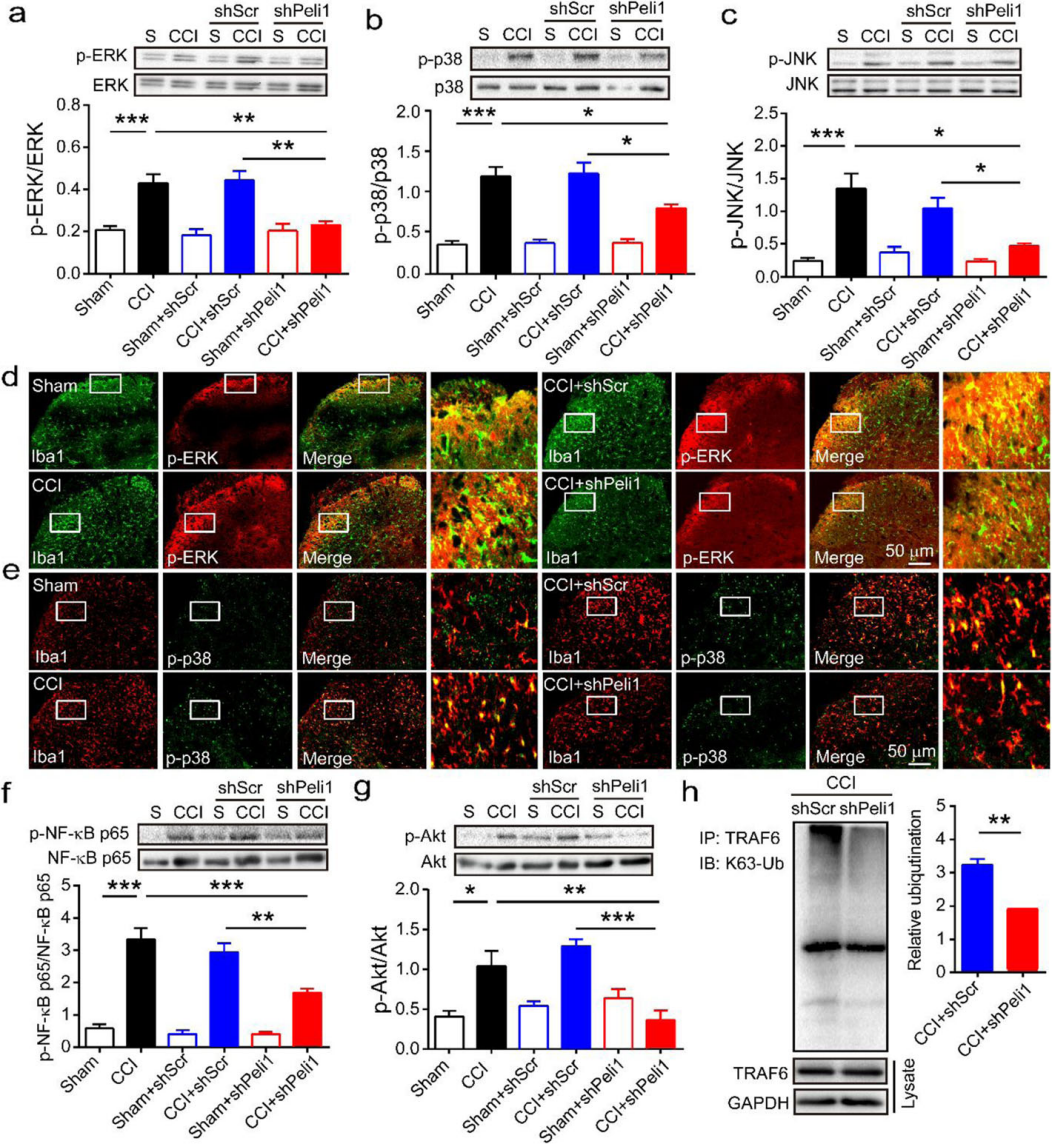
Spinal Peli1 is necessary for MAPK/NF-κB activation and TRAF6 ubiquitination after CCI. **a–c** Peli1 shRNA was given 3 days prior to the beginning of CCI. L4-L5 spinal cord tissues were collected at 7 days after CCI. Quantitative western blot analysis showing inhibitory effects of Peli1 shRNA on CCI-induced increased phosphorylation of ERK (**a**, *n* = 4–6), p38 (**b**, *n* = 4–5), and JNK (**c**, *n* = 4). One-way ANOVA [(**a**) *F*_5,21_ = 12.3; (**b**) *F*_5,20_ = 24.9; (**c**) *F*_5,18_ = 14.5]. **d**, **e** Double immunofluorescence staining showing lower colocalization of p-ERK (red) and p-p38 (green) with microglia marker Iba1 (red or green) by Peli1 shRNA in the ipsilateral dorsal horn at 7 days (scale bar 50 μm). **f**, **g** Peli1 shRNA suppresses CCI-induced increases in p-NF-κB p65 (**f**, *n* = 4–5) and P-Akt (**g**, *n* = 4–5) in the ipsilateral spinal cord on day 7 after injury. One-way ANOVA [(**f**) *F*_5,19_ = 42.8; (**g**) *F*_5,19_ = 10.2]. **h** Analysis of K63-linked ubiquitination of TRAF6 in the ipsilateral spinal cord that transfected with Peli1 shRNA or scrambled shRNA 3 days before the beginning of CCI. Tissues were collected at 7 days after CCI (*n* = 4). Results are expressed as the Mean ± SEM. *p < 0.05, **p < 0.01, ***p < 0.001 compared with indicated group

Finally, we defined the molecular mechanism underlying the regulation of neuropathic pain by Peli1. It is well documented that MAPK signaling plays an important role in neuropathic pain [33]. Additionally, MAPK signaling controls the release of proinflammatory cytokines in activated spinal cord microglia [34]. As shown in Fig. 6a–c, CCI caused great increases in ERK phosphorylation, p38 phosphorylation, and JNK phosphorylation respectively, compared with their respective sham controls. In contrast, blockade of Peli1 remarkably attenuated the expression of p-ERK, p-p38, and p-JNK activation by 46.4%, 32.7%, and 65.4% in the ipsilateral spinal cord, when compared with the respective CCI control. Injection of scrambled shRNA did not prevent the upregulation of p-ERK, p-p38, and p-JNK expression after CCI. Of note, double immunofluorescence staining further showed an intrathecal injection of Peli1 shRNA led to the attenuation of CCI-induced p-ERK and p-p38 accumulation in spinal cord microglia. However, intrathecal injection of scrambled shRNA did not reduce the accumulation of p-ERK and p-p38 in Iba1 in the ipsilateral dorsal horn (Fig. 6d, e).

It has been demonstrated that Peli1 is necessary for NF-κB activation [35], which is an important contributor to the development of neuropathic pain [36, 37]. Thus, we sought to examine whether the expression of phosphorylated NF-κB p65 would be changed after CCI. Western blot analysis showed that intrathecal injection of Peli1 shRNA led to a significant reduction of P-NF-κB p65 expression by 49.5% after CCI, when compared with CCI control. However, administration of scrambled shRNA did not attenuate CCI-induced increase in P-NF-κB p65 expression in the ipsilateral spinal cord (Fig. 6f). Peli1 is also reported to activate Akt [38, 39], which is an important signaling in the regulation of neuropathic pain [40, 41]. We found that knocking down Peli1 induced a significant reduction in Akt phosphorylation by 65.1% in the ipsilateral spinal cord, in comparison with scrambled shRNA control (Fig. 6g).

### Downregulation of Peli1 reduces ubiquitination of TRAF6 during CCI

To understand how Peli1 mediates CCI-induced activation of MAPK signaling and NF-κB, we examined the ubiquitination of TRAF6 that is an important signaling molecule regulating MAPK signaling and NF-κB [42]. Figure 6 h shows that knocking down spinal Peli1 significantly reduced K63-linked ubiquitination of TRAF6 in the ipsilateral spinal cord after CCI.

### Downregulation of Peli1 inhibits LPS-stimulated inflammatory reactions in BV2 microglial cells

**Fig. 7:**
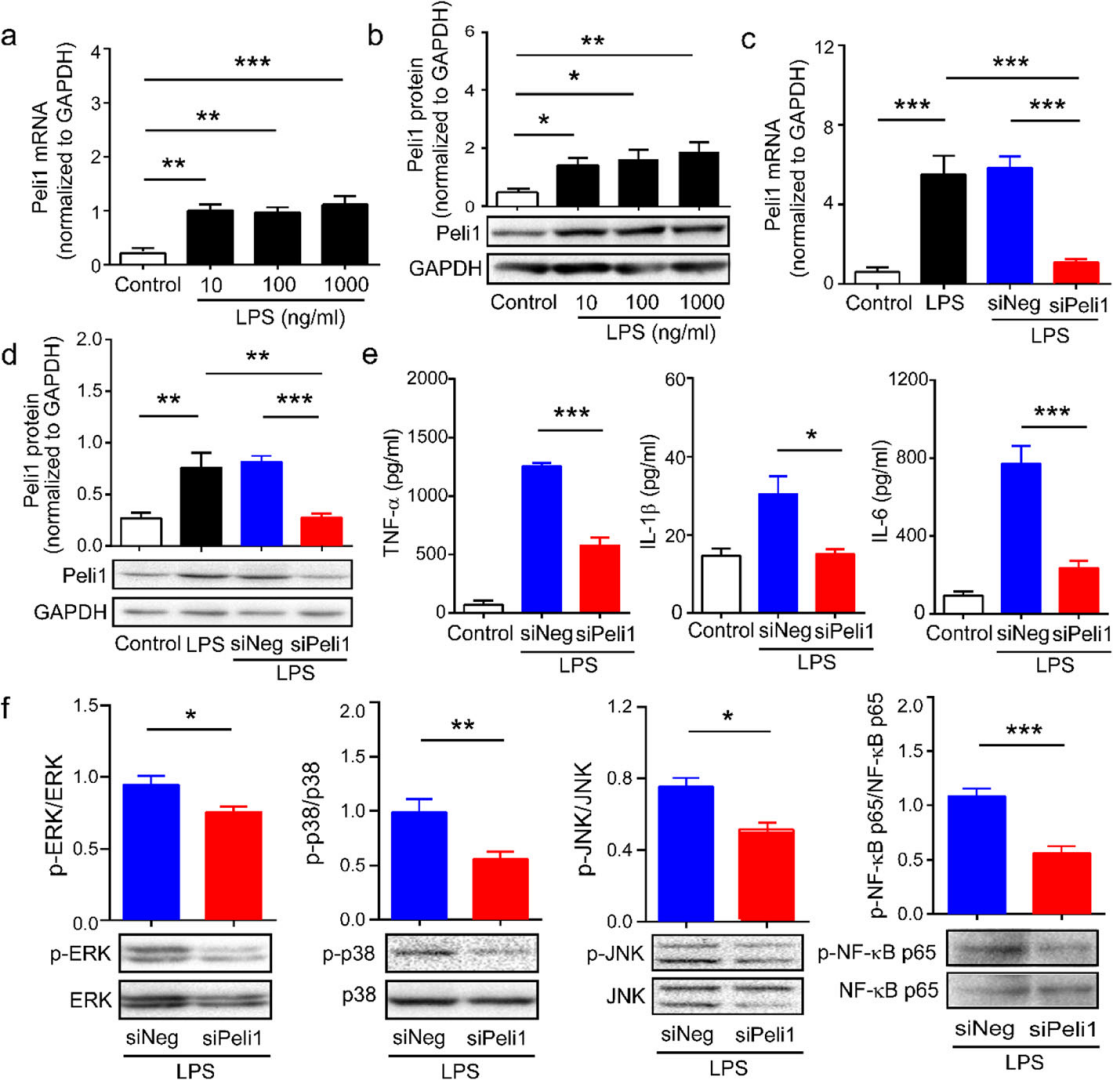
LPS-induced inflammatory reactions were suppressed by inhibition of Peli1 in BV2 microglial cells. **a**, **b** BV2 cells were pretreated without or with 10, 100, and 1000 ng/ml LPS for 2 h. Peli1 mRNA (**a**) and protein (**b**) levels were determined by qPCR and western blot respectively. One-way ANOVA [(**a**) *F*_3,14_ = 11; (**b**) *F*_3,11_ = 7.9]. **c–f** BV2 microglial cells were transfected with Peli1 siRNA or negative siRNA for 32 h before stimulated without or with LPS (100 ng/ml) for 2 h. Transfection of Peli1 siRNA suppresses LPS-induced increases in Peli1 mRNA (**c**) and protein (**d**) levels. One-way ANOVA [(**c**) *F*_3,13_ = 26.9; (**d**) *F*_3,17_ = 13.8]. (**e**) ELISA analysis showing TNF-α, IL-1β, and IL-6 release in BV2 microglia culture medium and the inhibitory effects of Peli1 siRNA on the release. One-way ANOVA (*F*_2,9_ = 134.8; *F*_2,8_ = 8.5; *F*_2,11_ = 35.1). (**f**) Western blot showing the inhibitory effects of Peli1 siRNA on the changes of phosphorylation level of MAPK and NF-κB p65 in cell lysates of BV2 cells were subjected to LPS stimulation. Results are expressed as the Mean ± SEM. *p < 0.05, **p < 0.01, ***p < 0.001 compared with indicated group

After demonstrating that CCI-induced increase in Peli1 contributes to microglial activation, we sought to examine a direct effect of Peli1 on BV2 microglial cells. We found that Peli1 mRNA and protein levels were significantly elevated in BV2 cells after LPS treatment (Fig. 7a, b). However, BV2 cells transfected with Peli1 siRNA exhibited significant reduction in LPS-induced increase in the expression of Peli1 (Fig. 7c, d). The results of ELISA showed that the massive secretion of TNF-α, IL-6, and IL-1β in LPS-stimulated BV2 cells were significantly attenuated by Peli1 siRNA (Fig. 7e). As expected, Fig. 7 f shows that Peli1 silencing significantly decreased the expression of p-ERK, p-p38, p-JNK, and p-NF-κB p65 in BV2 cells after LPS treatment. In addition, we observed that Peli1 shRNA attenuated LPS-induced activation of MAPK and NF-κB p65 as well as the production of TNF-α, IL-1β, and IL-6 in BV2 cells (Fig. S3).

### Downregulation of Peli1 in BV2 cells inhibits LPS-stimulated microglial activation

**Fig. 8:**
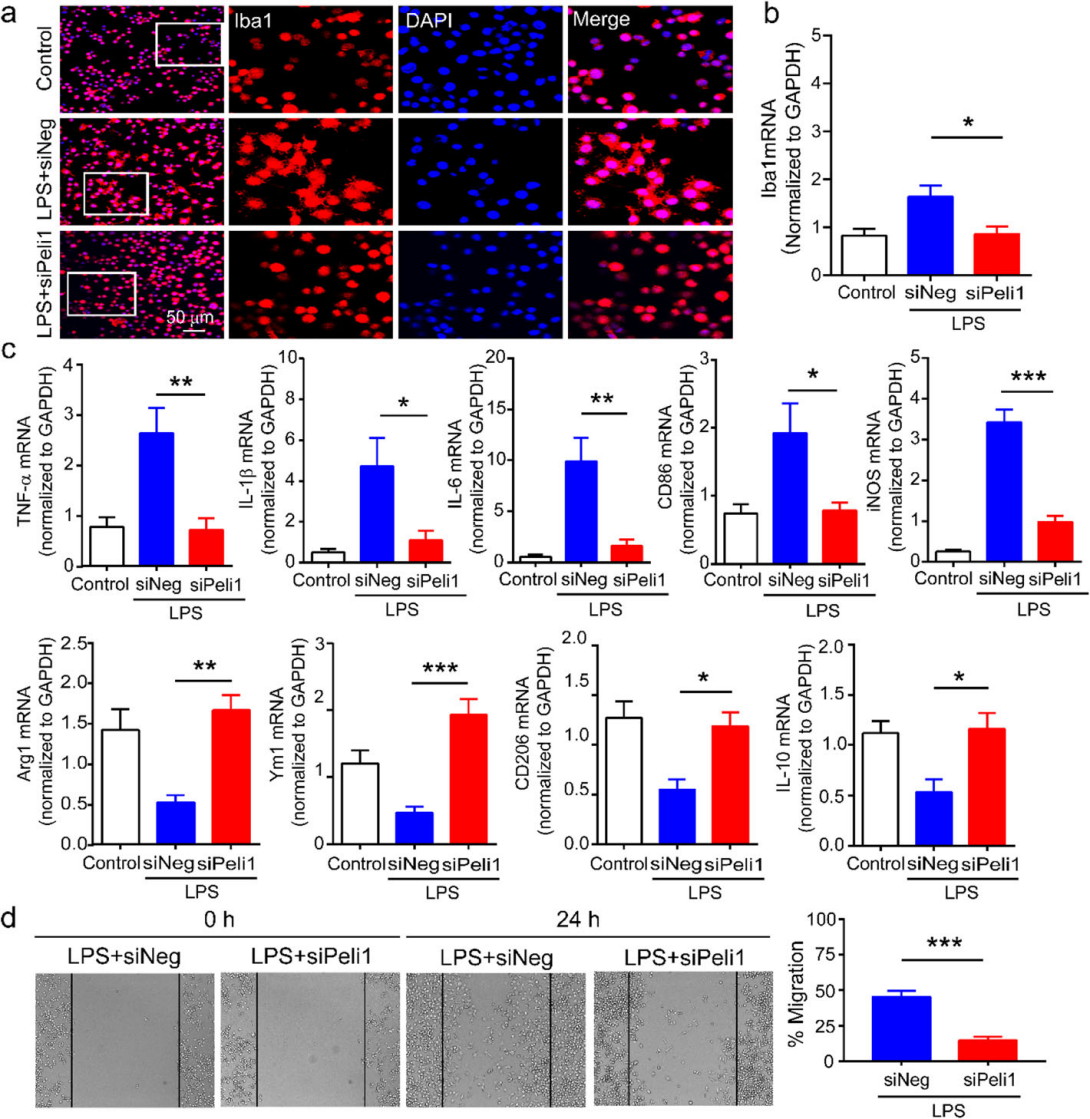
LPS-induced BV2 microglial activation was suppressed by inhibition of Peli1. **a** After LPS stimulation, transfected BV2 cells were fixed and labeled with DAPI (blue) and anti-Iba1 (red, scale bar 50 μm). **b** qPCR showing the inhibitory effects of Peli1 siRNA on the mRNA expression of Iba1 in BV2 cells after LPS treatment. One-way ANOVA (*F*_2,14_ = 5.9). **c** The effects of Peli1 on microglial M1 polarization markers (TNF-α, IL-1β, IL-6, CD86, iNOS) and M2 polarization markers (Arg1, Ym1, CD206, IL-10) mRNA expression in BV2 cells after LPS treatment were revealed by qPCR. One-way ANOVA (*F*_2,10_ = 10.9; *F*_2,9_ = 7.3; *F*_2,10_ = 15.2; *F*_2,12_ = 6.2; *F*_2,12_ = 67.0; *F*_2,9_ = 16.2; *F*_2,9_ = 8.4; *F*_2,9_ = 1.2; *F*_2,9_ = 6.6). **d** After 24 h of transfection, scratch assay in BV2 cells treated with 100 ng/ml LPS for 24 h. Transfection of Peli1 siRNA inhibits BV2 cells migration into the wound area. Representative images were taken before and 24 h after injury (× 4 magnification). Dashed line indicates the width of gap. Results are expressed as the Mean ± SEM. *p < 0.05, **p < 0.01, ***p < 0.001 compared with indicated group

We continued to examine the effect of Peli1 on the activation of BV2 cells assessed by Iba1 immunofluorescence in response to LPS challenge. Figure 8 a shows that Iba1 immunoreactivity was considerably decreased in Peli1 siRNA-transfected BV2 cells after LPS treatment. Moreover, Peli1 siRNA-transfected BV2 cells followed by LPS treatment displayed shorter branches compared with cells transfected with negative siRNA. The results of qPCR showed that Iba1 mRNA levels were significantly lower in the Peli1 siRNA group than that in the negative siRNA group after LPS stimulation (Fig. 8b). Furthermore, we found that knocking down Peli1 prevented LPS-induced M1 microglial polarization and promoted the M2 phenotype. Results showed that Peli1 siRNA inhibited the increase of M1 markers (TNF-α, IL-6, IL-1β, CD86, and iNOS) and enhanced the expression of M2 markers (Arg1, Ym1, CD206, and IL-10) after LPS treatment (Fig. 8c). In addition, we observed that Peli1 siRNA-transfected BV2 cells reduced the migration to the scratched area, indicating that Peli1 is involved in the migratory potential of microglia (Fig. 8d).

## Discussion

Microglia are the first responders and principal resident immune cells of the central nervous system [43]. A large body of work has demonstrated that microglial activation is the early event that mediates neuroinflammation in neuropathic pain [44, 45]. Therefore, a further understanding of microglial activation is necessary for expanding our knowledge regarding the pathogenesis of neuropathic pain. Our study was one of earliest evidences to demonstrate that E3 ubiquitin ligase Peli1 contributes to pain hypersensitivity. This is likely due to Peli1 that exerts a strong influence on spinal microglial activation. We found that Peli1 was significantly upregulated in the great majority of microglia in the spinal dorsal horn after neuropathic pain. The elevation of Peli1 was able to induce spinal microglial activation along with the production of TNF-α, IL-6, and IL-1β, probably through the enhancement of MAPK signaling and NF-κB activation. Additionally, Peli1 facilitated K63-linked ubiquitination of TRAF6 in the ipsilateral spinal cord during neuropathic pain. Taken together, our findings demonstrate that Peli1 is an important regulator in neuropathic pain, and therefore it may serve as a novel therapeutic target for neuropathic pain.

Peli is a newly identified family of E3 ubiquitin ligases, including Peli1, Peli2, and Peli3 [46]. Specifically, Peli1 is emerging as a microglia-specific regulator to participate in the pathophysiological process of experimental autoimmune encephalomyelitis (EAE) [19], subarachnoid hemorrhage (SHA) [24], and West Nile virus (WNV) encephalitis [23]. However, the possible role of Peli1 in neuropathic pain is not explored briefly. By using a CCI-induced neuropathic pain model, here we revealed a rapid and persistent increase of Peli1 expression in the spinal cord after nerve injury. We further observed that the elevated spinal Peli1 contributed to the initiation phase of neuropathic pain. Moreover, knockdown of spinal Peli1 by Peli1 siRNA produced a partial rather than prolonger reversal of existing CCI-induced neuropathic pain. It is possible that Peli1 siRNA may undergo degradation in the cytoplasm and fail to effectively decrease Peli1 expression, leading to cause a lack of stronger behavior effect. Our double immunofluorescent staining results additionally showed that Peli1 was expressed in the majority of spinal microglia in the dorsal horn. These data indicate Peli1 in spinal microglia may participate in neuropathic pain. Despite the fact that spinal Peli1 was expressed relatively low in neurons and astrocytes, we do not exclude the potential role of spinal Peli1 from neurons and/or astrocytes in neuropathic pain. Therefore, further research using cell-specific approaches to downregulate Peli1 expression is required to distinguish which cell types initiate Peli1-dependent regulation of neuropathic pain.

Activation of glial cells in the spinal cord plays a crucial role in the pathogenesis of neuropathic pain [3–6]. In vitro study shows that Peli1 is excessively expressed in microglia by TLR2 and TLR4 ligand [19]. Similar to this study, we found that Peli1 was increased in the ipsilateral spinal cord microglia, suggesting that Peli1 may be a potential trigger for microglial activation after nerve injury. Indeed, we found that Peli1 produced a significant increase of Iba1-positive microglial cells in the spinal dorsal horn following CCI, as well as the upregulation of CCI-induced mRNA expression of Iba1 and CD11b. Additionally, our in vitro results showed that Peli1 resulted in microglial activation in response to LPS stimulation. It is noted that activated microglia in the spinal cord is recognized as a major cellular source of proinflammatory cytokines, such as TNF-α, IL-6, and IL-1β, which further activate surrounding astrocytes and promote the hypersensitivity of dorsal horn neurons [47]. Here, we observed that upregulation of Peli1 promoted the production of TNF-α, IL-6, and IL-1β in the ipsilateral dorsal horn. In vitro data showed that downregulation of Peli1 strongly inhibited proinflammatory cytokines production in LPS-treated BV2 microglial cells. Furthermore, we found that Peli1 triggered astrocytic activation and enhanced neuronal activity in the spinal dorsal horn after CCI. Collectively, these results suggest that Peli1 may be an important regulator of neuroinflammation in neuropathic pain.

In the context of nerve injury, MAPK signaling including ERK, p38, and JNK as well as NF-κB have been shown to activate in the spinal dorsal horn [10, 11, 13, 36, 37]. Particularly, nerve injury causes the phosphorylation of ERK and p38 in the spinal-activated microglia [11, 13, 16]. Previous studies have demonstrated that Peli1 deficiency suppresses LPS-induced activation of MAPK signaling in primary microglia [19]. Yet another study suggests that Peli1 is not necessary to regulate IL-1-induced activation of MAPK signaling in HEK293 cells [20]. The inconsistency most likely reflects the use of different cell types. Here, we observed that knocking down spinal Peli1 significantly decreased CCI-induced MAPK phosphorylation. Immunofluorescence further showed that inhibition of Peli1 markedly decreased the expression of p-ERK and p-p38 in the spinal cord microglia. Peli1 additionally regulates TLR-mediated NF-κB activation [35]. We found that downregulation of Peli1 attenuated CCI-induced NF-κB activation in the ipsilateral spinal cord. These results indicate that MAPK signaling and NF-κB are downstream effectors of Peli1 in CCI-induced neuropathic pain. Therefore, spinal Peli1 is involved in the pathogenesis of neuropathic pain at least partially by triggering microglia-mediated inflammatory response through the activation of MAPK signaling and NF-κB.

TRAF6 acts as a key scaffold protein that is essential for the activation of MAPK signaling and NF-κB [42]. A RING domain of TRAF6 that confers E3 ubiquitin activity to mediate its own auto-ubiquitination through catalyzation of K63 ubiquitin chains [48]. Furthermore, K63-linked ubiquitination of TRAF6 activates MAPK signaling and NF-κB, leading to trigger the inflammatory responses [49]. Interestingly, recent evidence has shown that Peli1 facilitates ubiquitination of TRAF6, subsequently leading to MAPK signaling and NF-κB activation in the myocardium [21, 22]. Another study shows that Peli1 mediates TLR4/NF-κB signaling through ubiquitination of IRAK-TRAF6 complex in microglia [50]. Notably, TRAF6 has been implicated in neuropathic pain [51]. In this study, we found that Peli1 facilitated K63-linked ubiquitination of TRAF6 in the ipsilateral spinal cord after CCI, suggesting that Peli1 may mediate ubiquitination of TRAF6, thus promoting activation of MAPK signaling and NF-κB after CCI. Paradoxically, other evidence indicates that peli1 promotes K63-linked ubiquitination of cIAP and K48-linked ubiquitination of TRAF3 upon LPS stimulation in microglia, leading to MAPK activation [19]. These inconsistent results most likely reflect different functions of Peli1 in different pathological states.

## Conclusions

In summary, we demonstrated that Peli1 plays an important role in neuropathic pain and promotes neuroinflammation through MAPK/NF-κB signaling. Thus, Peli1 may serve as a novel strategy for the treatment of neuropathic pain.

## Supplementary information

**Additional file 1: Fig. S1** The expression of GFP in the spinal dorsal horn after intrathecal injection of lentiviral Peli1 shRNA. Representative images showing GFP (green) expression in the spinal dorsal horn 6 days after intrathecal injection of lentivirus vector. The staining of NeuN (red), GFAP (red), and Iba1 (red) on spinal sections expression GFP (Scale bar: 50 μm, *n* = 3).

**Additional file 2: Fig. S2** The expression of Peli1 in the spinal microglia, neurons, and astrocytes after intrathecal injection of lentiviral Peli1 shRNA following CCI. Representative images showing Peli1 (green) expression in the spinal microglia (Iba1, red), neurons (NeuN, red), and astrocytes (GFAP, red; Scale bar: 50 μm). Quantification of number of Peli1 positive cells showing the inhibitory effect of Peli1 shRNA on the decreased expression of Peli1 in microglia, neurons, and astrocytes in the spinal dorsal horn after CCI (*n* = 3). Results are expressed as the Mean ± SEM. *p < 0.05, **p < 0.01 compared with indicated group.

**Additional file 3: Fig. S3** The inhibitory effect of Peli1 shRNA on LPS-induced inflammatory reactions in BV2 microglial cells. **a** BV2 microglial cells were transduced with Peli1 shRNA or scrambled shRNA for 72 h before stimulated with LPS (100 ng/ml) for 2 h. Western blot showing the inhibitory effects of Peli1 shRNA on Peli1 expression, MAPK phosphorylation, NF-κB p65 activation in BV2 cells subjected to LPS stimulation. **b** ELISA analysis showing TNF-α, IL-1β, and IL-6 release in BV2 microglia culture medium. Results are expressed as the Mean ± SEM. *p < 0.05, **p < 0.01, ***p < 0.001 compared with indicated group.

## Data Availability

The data and materials supporting the conclusions of this study are available from the corresponding author upon reasonable request. The authors will take responsible for maintaining availability.

## Abbreviations

Arg1: Arginase 1
CCI: Chronic constriction injury
CD11b: Cluster of differentiation 11b
CD206: Cluster of differentiation 206
CD86: Cluster of differentiation 86
DMEM: Dulbecco modified eagle medium
ELISA: Enzyme-linked immunosorbent assay
ERK: Extracellular-regulated protein kinases
FBS: Fetal bovine serum
GAPDH: Glyceraldehyde-3-phosphate dehydrogenase
GFAP: Glial fibrillary acidic protein
GFP: Green fluorescent protein
Iba1: Ionized calcium binding adaptor molecule 1
IL-10: Interleukin 10
IL-1β: Interleukin 1β
IL-6: Interleukin 6
iNOS: Inducible nitric oxide synthase
JNK: c-Jun N-terminal kinase
LPS: Lipopolysaccharide
MAPK: Mitogen-activated protein kinase
NeuN: Neuronal specific nuclear protein
NF-κB: Nuclear factor kappa B
Peli1: Pellino1
PVDF: Polyvinylidene fluoride
PWL: Paw withdrawal latency
PWT: Paw withdrawal threshold
SDS-PAGE: Sodium dodecyl sulfate-polyacrylamide gel electrophoresis
shRNA: Short hairpin RNA
siRNA: Small interfering RNA
TNF-α: Tumor necrosis factor α
TRAF: Tumor necrosis factor receptor associated factor
Ym1: Chitinase-like protein 3
