# Supplementary figures and images for: Pellino1 regulates neuropathic pain as well as microglial activation through the regulation of MAPK/NF-κB signaling in the spinal cord
Source: J Neuroinflammation. 2020 Mar 14;17:83. doi: 10.1186/s12974-020-01754-z (PMC7071701; doi:10.1186/s12974-020-01754-z)

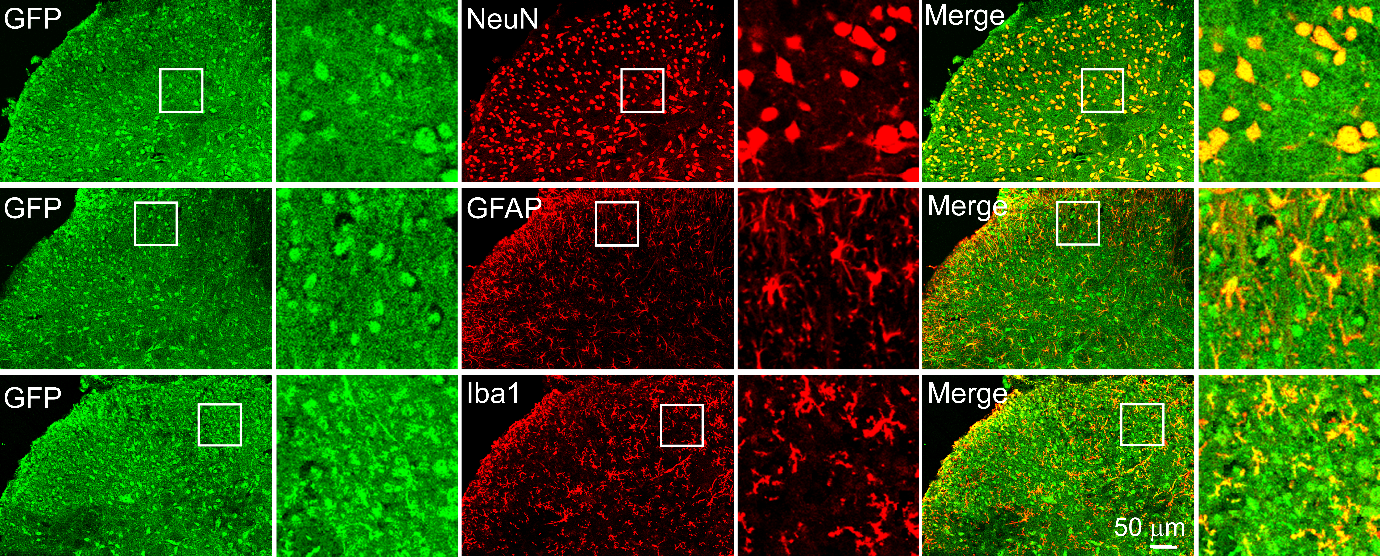

Supplement: Supplementary file 1 — Additional file 1: Fig. S1 The expression of GFP in the spinal dorsal horn after intrathecal injection of lentiviral Peli1 shRNA. Representative images showing GFP (green) expression in the spinal dorsal horn 6 days after intrathecal injection of lentivirus vector. The staining of NeuN (red), GFAP (red), and Iba1 (red) on spinal sections expression GFP (Scale bar: 50 μm, n = 3). [file 12974_2020_1754_MOESM1_ESM.docx]

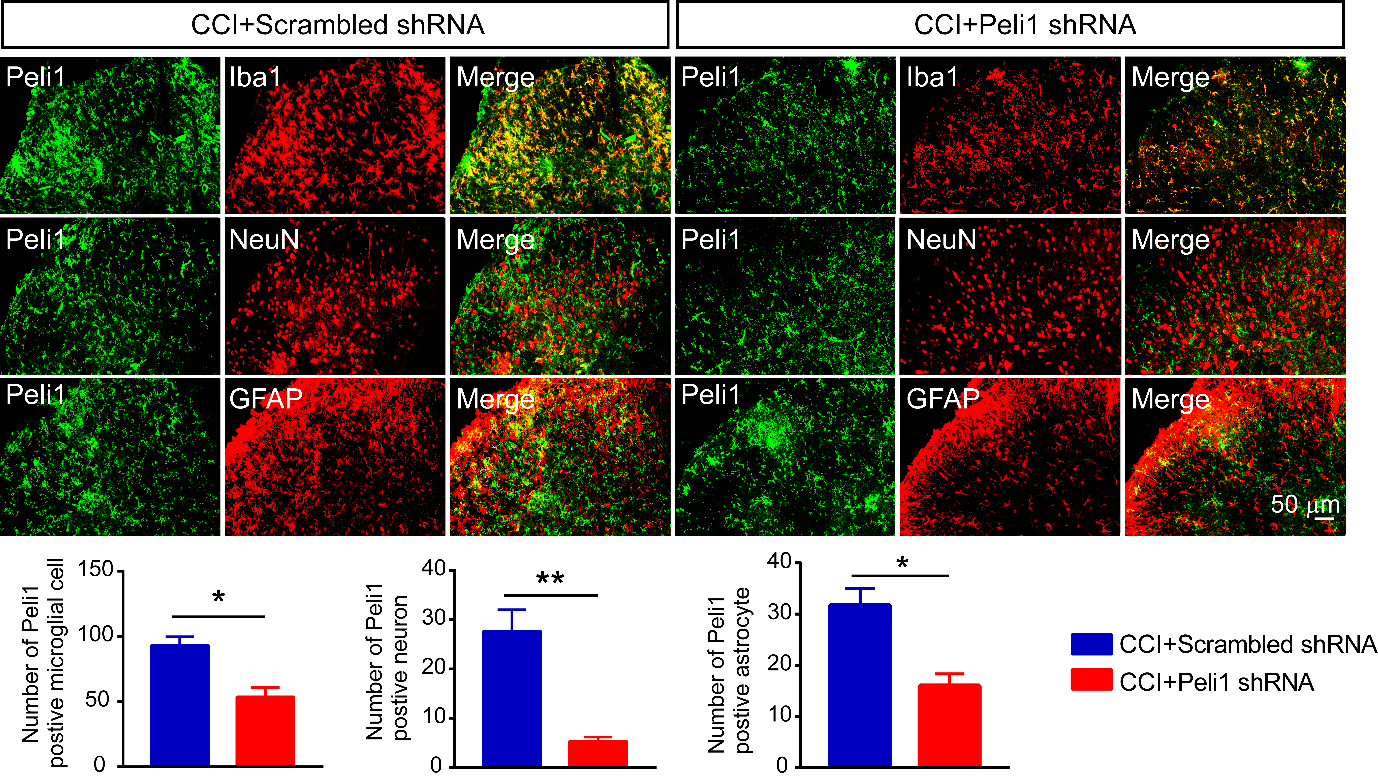

Supplement: Supplementary file 2 — Additional file 2: Fig. S2 The expression of Peli1 in the spinal microglia, neurons, and astrocytes after intrathecal injection of lentiviral Peli1 shRNA following CCI. Representative images showing Peli1 (green) expression in the spinal microglia (Iba1, red), neurons (NeuN, red), and astrocytes (GFAP, red; Scale bar: 50 μm). Quantification of number of Peli1 positive cells showing the inhibitory effect of Peli1 shRNA on the decreased expression of Peli1 in microglia, neurons, and astrocytes in the spinal dorsal horn after CCI (n = 3). Results are expressed as the Mean ± SEM. *p < 0.05, **p < 0.01 compared with indicated group. [file 12974_2020_1754_MOESM2_ESM.docx]

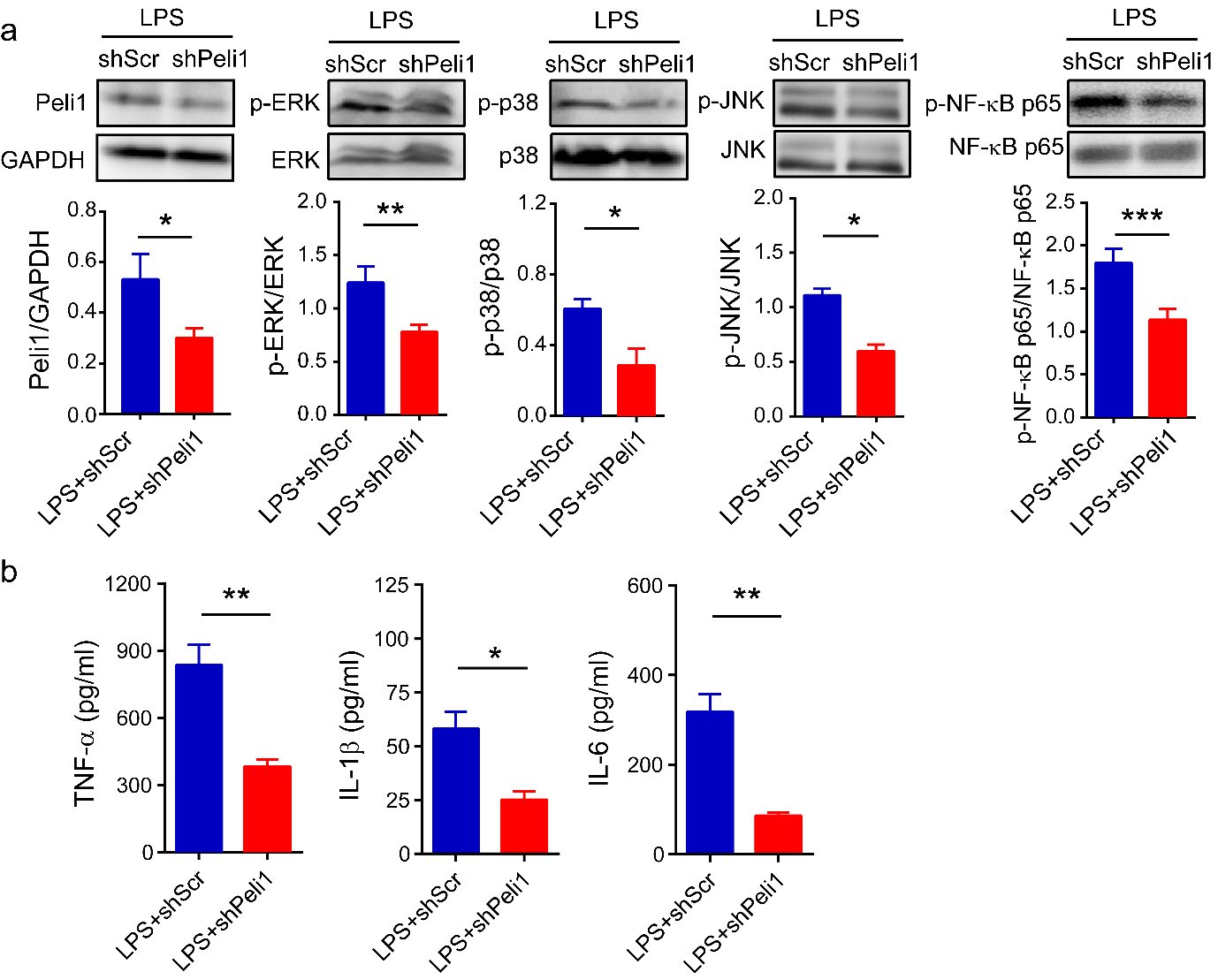

Supplement: Supplementary file 3 — Additional file 3: Fig. S3 The inhibitory effect of Peli1 shRNA on LPS-induced inflammatory reactions in BV2 microglial cells. a BV2 microglial cells were transduced with Peli1 shRNA or scrambled shRNA for 72 h before stimulated with LPS (100 ng/ml) for 2 h. Western blot showing the inhibitory effects of Peli1 shRNA on Peli1 expression, MAPK phosphorylation, NF-κB p65 activation in BV2 cells subjected to LPS stimulation. b ELISA analysis showing TNF-α, IL-1β, and IL-6 release in BV2 microglia culture medium. Results are expressed as the Mean ± SEM. *p < 0.05, **p < 0.01, ***p < 0.001 compared with indicated group. [file 12974_2020_1754_MOESM3_ESM.docx]
